# Global Longitudinal Strain Reduction With Apical Sparing in Cushing Syndrome-Related Heart Failure With Preserved Ejection Fraction (HFpEF): A Case Report

**DOI:** 10.7759/cureus.93445

**Published:** 2025-09-28

**Authors:** Abbas Rachid, Batoul Chaaban, Malek Mohammed, Ali Jibai, Hasan Kazma

**Affiliations:** 1 Department of Internal Medicine, Lebanese University, Beirut, LBN; 2 Department of Cardiology, Lebanese University, Beirut, LBN; 3 Department of Cardiology, Bahman University Hospital, Beirut, LBN; 4 Department of Nephrology, Bahman University Hospital, Beirut, LBN

**Keywords:** cushing’s disease, endogenous hypercortisolism, heart failure, heart failure cardiac amyloidosis, heart failure with preserved ejection fraction

## Abstract

We describe a case of a 56-year-old woman with a history of recurrent pituitary adenoma, not well followed, and known comorbidities of coronary artery disease, hypertension, and type 2 diabetes mellitus. She arrived with severely high blood pressure and signs pointing to hypercortisolism. Further evaluation revealed left ventricular hypertrophy, reduced global longitudinal strain, and preserved left ventricular ejection fraction, consistent with heart failure with preserved ejection fraction (HFpEF). Workup for amyloidosis was negative. This case highlights that chronic hypercortisolism may cause pathophysiological changes in the heart, leading to HFpEF, and may induce myocardial fibrosis and impaired myocardial mechanics, producing an echocardiographic pattern that can mimic infiltrative cardiomyopathy. Recognition of this overlap is crucial to avoid misdiagnosis and to ensure timely endocrine and cardiovascular management.

## Introduction

Hypercortisolism is defined as a clinical condition resulting from excessive tissue exposure to cortisol or other glucocorticoids. Sustained exposure ultimately leads to Cushing syndrome (CS), a well-established constellation of clinical manifestations arising from chronic endogenous or exogenous cortisol excess [1]. CS is associated with profound metabolic derangements that significantly increase cardiovascular risk, not only during the active phase of the disease but also persisting long after biochemical remission [2,3]. Cardiovascular complications, including premature atherosclerosis, coronary artery disease (CAD), heart failure, and cerebrovascular events, are major contributors to the excess mortality observed in CS compared with the general population [1,3]. Among these complications, arterial hypertension remains the most frequent cardiovascular disorder in patients with Cushing disease (CD) [4].

Although left ventricular (LV) systolic function is generally preserved in patients with CS, several studies have demonstrated that chronic cortisol excess induces structural and functional cardiac alterations, predisposing to major adverse cardiac events and the development of heart failure [5] In the broader context of chronic congestive heart failure, disease progression is tightly coupled with activation of neuroendocrine stress pathways, most notably the hypothalamic-pituitary-adrenal axis, which governs cortisol secretion [6]. Cortisol, a pivotal stress hormone, increases in response to physiological strain, and its sustained elevation contributes to adverse myocardial remodeling.

Heart failure with preserved ejection fraction (HFpEF), a chronic and progressive syndrome, exemplifies the deleterious effects of persistent myocardial stress. While overt heart failure is an uncommon complication of CS, when it does occur, it most often presents with preserved LV ejection fraction (LVEF) or with subclinical LV dysfunction [7]. Prior evidence has also linked CS to LV hypertrophy, diastolic dysfunction, and subtle systolic impairment, with many of these changes demonstrating reversibility upon normalization of cortisol levels [8].

This case is unique as it highlights the interplay between CS and cardiac amyloidosis, emphasizing their overlapping yet distinct echocardiographic features. Global longitudinal strain (GLS), a measure of myocardial deformation, is particularly useful for differentiating these conditions and reveals subtle differences in strain patterns between the two.

## Case presentation

A 56-year-old woman with a significant past medical history of recurrent pituitary macroadenoma, treated with two prior surgical resections, the most recent five years earlier without subsequent follow-up, CAD, long-standing hypertension, and type 2 diabetes mellitus, presented to the emergency department with hypertensive urgency.

On arrival, she presented with a hypertensive crisis, with blood pressure measured at 200/110 mmHg, associated with severe cephalalgia, without syncope, visual changes, or focal neurological deficits. An MRI Brain demonstrated no evidence of acute intracranial hemorrhage or mass effect (Video 1). Initial laboratory testing showed normal complete blood count, renal function, and serum electrolytes. On physical examination, she exhibited characteristic Cushingoid stigmata, including rounded moon facies, central adiposity, and bilateral lower-extremity pitting edema.

**Video 1:** T2-FLAIR MRI of the brain shows no evidence of acute hemorrhage, mass effect, or acute ischemic stroke T2-FLAIR: T2-Weighted Fluid Attenuated Inversion Recovery

She was commenced on intensive antihypertensive therapy, including spironolactone, clonidine, telmisartan, carvedilol, amlodipine, and intravenous furosemide (20 mg, subsequently escalated to 40 mg). Given her clinical appearance and history of pituitary disease, an endocrine evaluation was undertaken. An overnight dexamethasone suppression test revealed an unsuppressed morning cortisol of 360 nmol/L, consistent with hypercortisolism.

**Figure 1:**
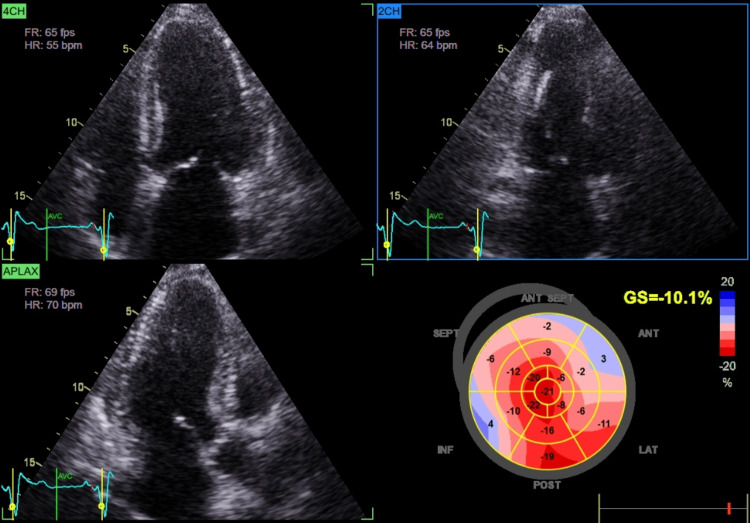
Transthoracic echocardiography demonstrating reduced global longitudinal strain (-10%) consistent with preserved EF (60%) EF: Ejection Fraction

Cardiac assessment supported a diagnosis of HFpEF. Transthoracic echocardiography demonstrated preserved left ventricular ejection fraction (60%), impaired GLS (-10%), and mild concentric left ventricular hypertrophy (Figure 1; Video 2). 

**Video 2:** TTE with strain analysis: reduced GLS (-10.1%) despite preserved LVEF (60%) TTE: Transthoracic Echocardiography; GLS: Global Longitudinal Strain; LVEF: Left Ventricular Ejection Fraction

Workup for alternative causes of HFpEF, including renal impairment and infiltrative cardiomyopathy, was unremarkable; both serum and urine protein electrophoresis with immunofixation excluded amyloidosis.

Magnetic resonance imaging of the pituitary revealed recurrence of the macroadenoma. The patient was referred to neurosurgery for consideration of repeat resection, and glucocorticoid-sparing medical therapy was initiated. During hospitalization, her blood pressure was gradually stabilized, diuretic therapy improved signs of congestion, and her functional status returned to near baseline with restored mobility (Video 3).

**Video 3:** Sagittal T1-weighted MRI shows a pituitary macroadenoma, characterized by an enlarged pituitary mass extending superiorly into the suprasellar cistern with mass effect on the optic chiasm

## Discussion

Epidemiology and clinical significance

CD is a severe endocrine disorder characterized by chronic exposure to excess glucocorticoids. Patients with CD have a two- to fivefold higher mortality compared with the general population, predominantly due to cardiovascular complications [4]. Chronic hypercortisolism is associated with systemic hypertension, left ventricular hypertrophy (LVH), diastolic dysfunction, and accelerated atherosclerosis, increasing the risk of myocardial ischemia and heart failure. While these cardiovascular manifestations are common, the development of isolated dilated cardiomyopathy (DCM) in the absence of other major comorbidities is rare but clinically noteworthy [9].

Pathophysiology of cardiac involvement

Chronic glucocorticoid excess contributes to cardiovascular remodeling via multiple mechanisms. Persistent hypertension and metabolic disturbances promote LVH and diastolic dysfunction. Additionally, glucocorticoid excess induces endothelial dysfunction, insulin resistance, and myocardial fibrosis, impairing ventricular compliance and predisposing to HFpEF [1,6]. Advanced echocardiographic techniques, such as GLS, can detect subclinical systolic dysfunction before overt reductions in LVEF [6]. In our patient, preserved LVEF (60%) coupled with markedly reduced GLS (-10%) and concentric LVH was consistent with HFpEF secondary to chronic cortisol excess, further supported by clinical signs of volume overload such as edema and severe hypertension [7].

Apical sparing and mimicking amyloidosis

An important observation in this case was relative apical sparing despite markedly reduced GLS, a strain pattern classically associated with cardiac amyloidosis [10]. Although infiltrative disease was excluded (negative serum and urine protein electrophoresis with immunofixation), this overlap illustrates how hypercortisolism-induced remodeling can phenocopy amyloidosis on imaging. Recent work has shown that hypercortisolism, beyond metabolic derangements, impairs myocardial mechanics and contractile efficiency [11]. Thus, patients with atypical strain findings should undergo careful endocrine evaluation to avoid misdiagnosis. Ultimately, the recognition that hypercortisolism may produce amyloid-like echocardiographic signatures has both diagnostic and management implications. It broadens the differential diagnosis of HFpEF and stresses the need for a multidisciplinary approach involving endocrinology and cardiology to prevent misdiagnosis and ensure tailored therapy.

Dilated cardiomyopathy in CS

Although uncommon, DCM with severe LV systolic dysfunction has been described in CS. Frustaci et al. reported eight cases of hypercortisolism due to adrenal adenoma among 473 patients with DCM (1.7%), all presenting with LVEF <30% and symptomatic heart failure. Endomyocardial biopsy revealed cardiomyocyte hypertrophy, interstitial fibrosis, and myofibrillolysis, distinct from idiopathic DCM and valvular disease controls. Follow-up biopsies in three patients one year post-adrenalectomy demonstrated substantial regression of these changes, highlighting the reversibility of glucocorticoid-induced myocardial injury [12].

Although not assessed in our patient, prior studies have implicated atrogin-1 in CS-related myocardial remodeling. At the molecular level, upregulation of atrogin-1, an E3 ubiquitin ligase expressed in skeletal, smooth, and cardiac muscle, was observed in CS-associated DCM compared with idiopathic DCM and controls [13]. Atrogin-1, implicated in skeletal muscle atrophy and sarcopenia, facilitates proteasomal degradation of intracellular proteins. Its overexpression in cardiomyocytes contributes to glucocorticoid-mediated myocardial remodeling. Importantly, atrogin-1 expression declined significantly following surgical correction of cortisol excess, paralleling improvements in cardiac structure and function. This reversibility mirrors recovery seen in glucocorticoid-induced skeletal myopathy and underscores the unique potential for cardiac recovery in CS-related DCM [9].

Clinical implications and differential diagnosis

This case underscores the multisystem burden of endogenous hypercortisolism, with particular cardiovascular susceptibility [1,6]. Chronic cortisol excess should be considered in the differential diagnosis of HFpEF, particularly when conventional risk factors coexist with systemic features such as central obesity, moon facies, and proximal myopathy [8]. Secondary causes of HFpEF, including cardiac amyloidosis, were excluded, supporting hypercortisolism as the primary etiology. Recognizing CS as a reversible contributor to myocardial dysfunction has important clinical implications, as timely endocrine intervention can improve cardiac function, lower blood pressure, and potentially prevent progression to irreversible myocardial remodeling.

Left ventricular hypertrophy and structural remodeling

Electrocardiographic and echocardiographic studies have characterized the cardiac phenotype in patients with CS. In a cohort of 12 consecutive patients, most had concomitant hypertension (11/12) and diabetes mellitus (7/12). Preoperative ECGs commonly demonstrated high-voltage QRS complexes (10 patients) and T-wave inversions (7 patients), indicative of LV strain. Echocardiography revealed LVH in nine patients, all exhibiting asymmetric septal hypertrophy. Interventricular septal thickness ranged from 16 to 32 mm, with septal-to-posterior wall ratios from 1.33 to 2.67. Compared with essential hypertension or primary aldosteronism, CS patients exhibited more pronounced LVH and a higher prevalence of asymmetric septal hypertrophy, suggesting a unique glucocorticoid-mediated remodeling pattern [13].

Postoperative follow-up in nine patients demonstrated normalization of ECG abnormalities, decreased septal thickness, and resolution of asymmetric septal hypertrophy in all but one patient, highlighting the partial reversibility of LVH following correction of hypercortisolism. The pronounced septal thickening relative to the posterior wall implies that excessive cortisol exposure, beyond hemodynamic effects of hypertension, contributes significantly to myocardial remodeling [13].

Impact of disease duration on concentric remodeling

Fallo et al. evaluated 18 patients with CS compared with 18 matched controls, adjusting for sex, age, body size, blood pressure, and duration of hypertension. Eleven participants in each group were hypertensive. Echocardiography revealed elevated relative wall thickness (RWT >0.45) in 11 patients with CS (five normotensive, six hypertensive) versus two hypertensive controls. Left ventricular mass index was abnormal in three CS patients and in four hypertensive controls, while systolic function was preserved in all participants [14].

No correlation was observed between RWT and either blood pressure or urinary cortisol levels in patients with CS. Instead, RWT correlated significantly with disease duration, indicating that prolonged exposure to glucocorticoid excess, rather than hormone levels or hemodynamic load, is the primary determinant of concentric LV remodeling. Postoperative echocardiography showed normalization of RWT in five of six patients previously affected, reinforcing the concept of reversible myocardial structural changes following correction of hypercortisolism [14].

## Conclusions

CS represents a rare but clinically important etiology of heart failure with preserved ejection fraction and, less commonly, dilated cardiomyopathy. Chronic hypercortisolism promotes systemic hypertension, LVH, diastolic dysfunction, myocardial fibrosis, and remodeling that may mimic infiltrative cardiomyopathies such as amyloidosis on echocardiography. GLS with apical sparing, although typically associated with amyloidosis, may also occur in cortisol-induced cardiomyopathy. Advanced imaging, including GLS, can detect subclinical myocardial impairment before overt systolic dysfunction develops. Notably, cardiac structural and functional abnormalities may partially or completely reverse following normalization of cortisol levels, highlighting the importance of early recognition and timely endocrine intervention. Clinicians should maintain a high index of suspicion for hypercortisolism in patients presenting with unexplained LVH, HFpEF, or atypical DCM, particularly when systemic features of CS are present. Future studies are needed to better characterize strain patterns in endocrine cardiomyopathies and to refine imaging-based algorithms for early detection.
